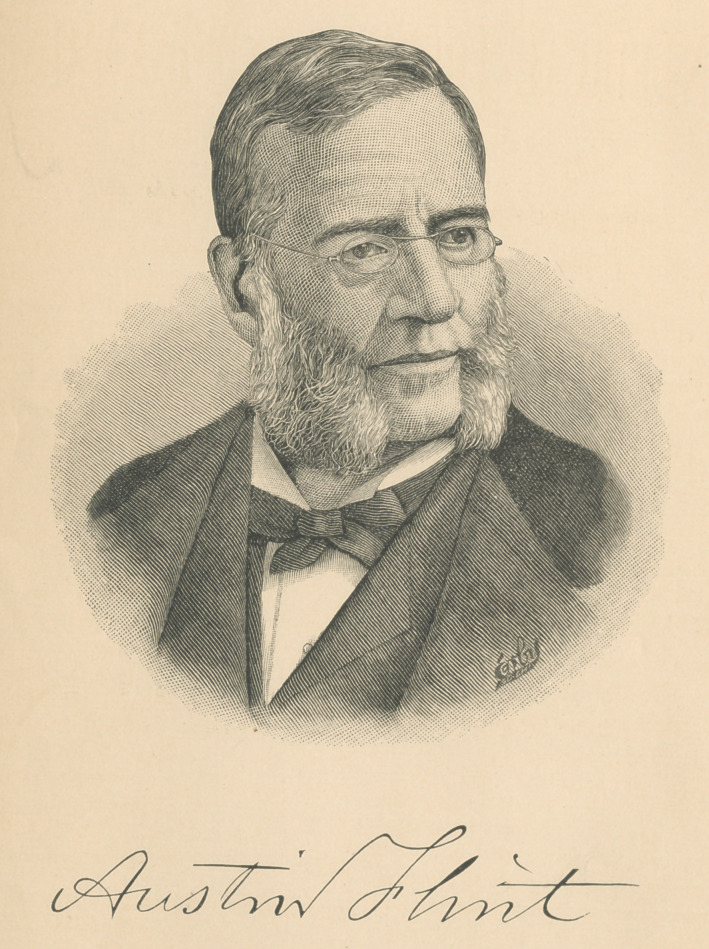# Antisepsis in Abdominal Operations

**Published:** 1887-07

**Authors:** 


### The Medical Journal and Examiner.

Vol. LV. JULY, 1887. No. 1.

### ANTISEPSIS IN ABDOMINAL OPERATIONS.

*SYNOPSIS OF SERIES OF BACTERIOLOGICAL STUDIES BY DR. CHRISTIAN FENGER AND DR. BAYARD HOLMES.

THESE investigations were undertaken to determine how far the necessary aseptic conditions had been secured and maintained in the abdominal sections performed by Dr. Christian Fenger. One case of another operator is brought in to compare less thorough antiseptic precautions.

In order to estimate the results of these researches, we must know what preparations were made for the operation on the part of each concerned.

### THE PREPARATION OF THE OPERATING ROOM.

In the Emergency Hospital and in the County Hospital, the walls and the floor and all the furniture were thoroughly washed with a 1-1000 sublimate solution on the day before the operation. The cracks about the windows and doors were stuffed with cotton, and the room closed to every one except the nurse that made the preparations.

To test the condition of the atmosphere in this room in the Emergency Hospital, four plates of gelatin were exposed for 48 hours on the operating table, August 24 and 25, 1886. After six days' incubation in the moist chamber, from eight to twelve colonies of all kinds appeared on each square inch of surface. Most of these were moulds, which grew very rapidly; some were micrococci and some bacilli. As less than twelve colonies developed to the square inch, it is probable, if the plates were exposed only an hour instead of 48 hours, not more than one colony would be found on each four square inches. So that the danger of atmospheric infection from falling germs would be very slight under similar conditions. The danger would, no doubt, be increased by the movements of the assistants, and the increased circulation of air through the difference in temperature of the external and internal atmosphere when the room was in use.

### THE PREPARATIONS OF THE OPERATOR AND ASSISTANTS.

Each took a sublimate bath (1-2000) and put on sterilized cotton suits. The

*This paper was read by Dr. Holmes before the Chicago Gynæcological Society, April 12, and the tubes containing the specimens exhibited.

hands and arms were then washed five minutes with warm water and green soap, and scrubbed with a brush, and then washed half a minute in a 1-1000 sublimate solution. The patient received substantially the same treatment.

### THE PREPARATION OF THE SPONGES, SILK, INSTRUMENTS, GAUZE AND WATER.

The sponges were those prepared by Schorse, cf Milwaukee.

The silk was boiled an hour in a 5 per cent. carbolic acid solution, and in some cases afterwards immersed in a solution of iodoform and ether and again sterilized by moist heat in a bottle stopped with cotton. This was done by placing the bottle in a pail containing an inch of water and boiling for an hour. The instruments were boiled an hour in a 5 per cent carbolic solution on the day before the operation and then dried. On the morning of the operation they were again boiled for a few minutes in a similar solution and placed in trays of sterilized water for use.

The water was sterilized by boiling in large tin cans, each holding 2 or 3 gallons, for an hour or more on three successive days. The cans had tin covers put on over a rim of cotton to stop the crack between itself and the can.

The culture medium used for these investigations was sterilized, alkaline, peptonized beef tea gelatin. Most bacteria will grow in this medium at the temperature of a living-room. Answers have been sought through these investigations to the following questions :

I.—*Are the sponges sterile when ready for use?*

After the sponges had been rinsed out in sterilized water three times, the assistant cut off small pieces from each of the sponges to be used with scissors, and they were put in a tube of liquified gelatin beef broth. This tube contains three such pieces of sponge surrounded by the clear solid gelatins.

*Out of twenty-five sponges from seven operations only a single sponge was found infected with a single colony.*

In this tube you will see the small triangular colony in the lower part of the upper sponge. With a small magnifying glass it seems to be a group of four or five spherical colonies in a cluster.

This sponge was used in operation xx., Nov. 30, sarcoma of the ovary, Emergency Hospital.

It would appear from this that the sponges are sterile at the beginning of the operations; and, if sterile, then of course aseptic.

II.—*Is the silk sterile?*

Five or six pieces of silk were usually taken from as many needles, and an inch cut off from each and put in a single tube of gelatin.

More than thirty such pieces were examined from nine operations, and not a single colony developed.

*In no case was the silk infected at the beginning of the operation.*

III.—*Is the cat gut sterile ?* (*Schorse's carbolized cat gut.*)

Several pieces at four operations were examined. In one case only did any colonies develop. In this tube you see two pieces of cat gut at the bottom of the clear gelatin. Clinging to the side of one piece you can discover a small spherical white colony, and a little distance from it in the gelatin another similar colony. This cat gut was from a new bottle of carbolized cat gut used in operation 5, in a private house in the country. It is difficult to say how significant their presence is. They might arise from any one of following causes :

1. Imperfect primary sterilization of the cat gut.

2. Infection by floating germ or germs from the hands of the assistant when unwinding and cutting off the pieces.

3. Infection through transportation to and from the country.

4. Imperfect sterilization of the nutrient medium.

It is my own opinion that it is from infection through the second above named causes.

Thus out of over thirty tubes, containing over sixty pieces of material taken before the operations, only *two* pieces were found infected with *three* colonies. This would indicate that the precautions taken are very successful at the beginning of the operations.

IV.—*Are the sponges sterile after they have been used?*

At the end of each operation, small pieces of each of the sponges used were cut off and placed in gelatin in the same manner as at the beginning. They were usually stained with blood, and sometimes had pieces of the contents of the cysts clinging to them. Thirty pieces from eight operations were thus examined. In this tube passed around, which contains two pieces of sponge from the last operaation, No. 8, you see numerous colonies on the side of the upper sponge. They are spherical and whitish, and do not liquefy the gelatin. The following is the list of the sponges infected:

Operation 1. 5 sponges examined. 1 infected.

Operation 2. 2 sponges examined. 1 infected.

Operation 5. 4 sponges examined. 1 infected.

Operation 8. 2 sponges examined. 1 infected.

The sponges were generally sterile at the close of the operation, even though most of them had come in contact with the skin of the abdomen and the contents of the cysts.

It may seem strange that the sponges used in operation 4, pyo-salpinx, in which the cyst burst into the abdomen, in tearing it away from its adhesions, did not develop any colonies. Five sponges were examined, and all remained apparently sterile. From the pus in this cyst, cultures were made in solid blood serum with the growth of a small micrococcus usually in the so-called diplococcus form, but this microbe would not grow in gelatin beef tea.

V.—*Is the silk sterile at the close of the operation and after it has been used as sutures?*

Out of twenty pieces of silk, often cut from the ends of abdominal sutures, only a single piece was infected with a single coccus form, viz.: one of the two pieces taken from operation 1.

Over fifty pieces of material, after being used in operations, and only five pieces—4 sponges and 1 piece of silk—were found infected. It does seem, therefore, that the sponges and silk may be maintained sterile, so far as any germs that will grow in nutrient gelatin are concerned, even to the end of a long operation.

In marked contrasts to these results appear those from an operation performed by another operator who kindly allowed similar examinations. The details of the preparations were given for publication.

The operating room was well washed with soap and water, both walls and floors. It was in a new house which had never contained a sick person.

The sponges were part new and part old, having been used in a previous abdominal section. After that operation they had been soaked a day in a strong solution of bicarbonate of soda, and washed out in a 5 per cent. solution of carbolic acid and hung away in a bag. On the day before the operation all the sponges were boiled in a porcelain kettle for more than an hour in a 2½ per cent. solution of carbolic acid, and put into a jar and taken to the operating room. The silk was boiled and carried in the same jar. The operator took a bath and put on perfectly clean clothes on the morning of the operation. The assistants were instructed to do the same. The hands and arms of the assistants were washed in soap and water and then in a sublimate solution 1-1000.

The material examined consisted of four sponges and two pieces of silk before the operation, after the sponges were rinsed out, and the needles threaded, and of two sponges after the operation and several inches of the thread used. All the material was infected except one piece of silk examined at the beginning of the operation. Every sponge had at least one colony of the hay bacillus, and one sponge after use showed more than 50 small white colors in the clear gelatin in the upper part of the tube.

What influence the asepsis of the material has on the results of the operations as to death or recovery, is a question far beyond the scope of these investigations. It would require a large statistical material of well observed cases, and more work than could be done by one observer. But it may be safe to conclude that it is desirable to work through an abdominal operation with perfect asepsis everywhere, if such a thing is possible.

The above investigations have shown that such perfect asepsis can be attained. Thus if we are ignorant of the extent of danger from non-sterile material, we are hardly justified in trusting to the innocence or inocuousness of such an uncertainty while we can have the asepsis of the material an absolute guaranty against the dangers of infection.

OPERATIONS PERFORMED BY DR. CHRISTIAN FENGER.

| No. Name of Operation and Date. | Complications. | Bacteriological Results. | Results as to Death or Recovery. |
|---|---|---|---|
| 1. Demoid of Ovary. Emergency Hospital. Nov. 9, 1886. | Bursting of cyst into the abdomen during operation. Irrigation. | 1 sponge and 1 piece of silk infected after operation. 4 tubes used to examine 12 pieces of material. | Recovery. Development of properitonecal abscess, which was opened in the sixth week. Complete recovery. |
| 2. Cysto Sarcoma of left ovary. Emergency Hospital. Nov. 30, 1886. | Solid movable tumor 6 inches in diameter, only slightly adherent to abdominal organs. Metastatasis in peritoneum. Some acites present. Drainage. | 6 tubes used to examine 11 pieces of material. 1 sponge before use and one sponge after use infected, each with a single colony. | Recovery. |
| 3. Rad cal Operation for Hernia. Emergency Hospital. Jan. 18, 1887. | | 8 tubes used to examine silk and sponges and cat gut. All sterile. | Recovery. |
| 4. Pyo-salpinx. Emergency Hospital. Jan. 19, 1887. | The Sac, adhering on all sides. Ruptured in removal. Irrigation of abdomen. | 14 tubes of nutrient gelatin used. All sterile. *Contents of cyst planted in solid human blood. Serum developed diplococci of very small size, which do not grow in gelatin,* probably gonococci. | Death from acute sepsis within 48 hours. |
| 5. Double Demoid. Private House. Jan. 24, 1887. | The tumor of the left side had ruptured twenty years before, and produced an almost fatal peritonitis (?) This tumor was now large and adherent all around. The tumor on the right side was small and free. | 9 tubes used with 25 pieces of material. One piece of cat gut infected with 2 colonies. One sponge infected with a single colony. | Death from shock within 12 hours. |
| 6. Cyst of ovary. Emergency Hospital. Feb. 3, 1887. | 7 tubes used to examine 10 pieces of material. All sterile. | | Recovery. |
| 7. Cyst of ovary. Emergency Hospital. March 22, 1887. | | 2 tubes used to examine silk and sponges after operation only. All sterile. | Recovery. |
| 8. Malignant Cyst of broad ligament. County Hospital. March — 1887. | | 4 tubes used. All sterile. | Death from uræmia on the 5th day. Autopsy. Atrophic and dilated kidneys. |
| 9. Proliferating Cystoma of ovary. Emergency Hospital. March 24, 1887. | Large and adherent. Burst during removal. Irrigation of abdomen to remove cyst contents. Drainage. | 5 tubes used, 12 pieces of material. Only one sponge infected with numerous colonies. | Death after 36 hours. Autopsy after 6 hours. Thrombosis of right ventricle. Bloody serum found in the peritoneal cavity in small quantities was added to nutrient gelatin. It remained sterile after two weeks' incubation. |

CONTRASTED CASE PERFORMED BY ANOTHER SURGEON IN WHICH LESS SUCCESSFUL ANTISEPTIC PRECAUTIONS WERE USED.

| | | | |
|---|---|---|---|
| Cysto-sarcoma of ovary. Private house. Feb. 22, 1887. | Very large, and adherent to omentum and abdominal wall. Drainage by means of two rubber drainage tubes. | 9 tubes used for silk, and sponges all infected except one, containing silk, before the operation. The sponges, after use, contained many 50-100 colonies. Each of the eight infected tubes had at least one colony of the Hay Bacillus. | Death on the third day. No autopsy. At each daily dressing there was evidence of some oozing from the drainage tubes. |

## Figures and Tables

**Figure f1:**